# Real-world smartphone-based point-of-care diagnostics in primary health care to monitor HbA1c levels in people with diabetes

**DOI:** 10.1038/s43856-025-00743-8

**Published:** 2025-02-05

**Authors:** Sabrina Rhode, Lisa Rogge, Marthoenis Marthoenis, Till Seuring, Hendra Zufry, Till Bärnighausen, Hizir Sofyan, Jennifer Manne-Goehler, Sebastian Vollmer

**Affiliations:** 1 Heidelberg Institute of Global Health, Heidelberg University, Heidelberg, Germany; 2 University of Göttingen, Centre for Modern Indian Studies - CeMIS, Göttingen, Germany; 3 Department of Ophthalmology, University of Luebeck, Luebeck, Germany; 4 Friedrich- Alexander- Universität Erlangen- Nürnberg, Institute of Economics, Erlangen, Germany; 5 Leibniz University Hanover, Lower Saxony, Hanover, Germany; 6 Department of Psychiatry and Mental Health Nursing, Universitas Syiah Kuala, Darussalam, Banda Aceh, Aceh Indonesia; 7 Luxembourg Institute of Socio-Economic Research - LISER, Esch/Alzette, Luxembourg; 8 Zoeinal Abidin Hospital, Darussalam, Banda Aceh, Aceh Indonesia; 9 Syiah Kuala University, Darussalam, Banda Aceh, Aceh Indonesia; 10 Division of Infectious Diseases, Brigham and Women’s Hospital, Harvard Medical School, Boston, MA USA

**Keywords:** Laboratory techniques and procedures, Diabetes, Public health

## Abstract

**Background:**

The lack of accurate and affordable monitoring of glycated hemoglobin (HbA1c) is a common issue among patients with diabetes in low- and middle-income countries. We aimed to test a tablet- and smartphone-based point-of-care (TSB POC) device against a local laboratory-based measure of HbA1c for monitoring diabetes under real-world conditions.

**Methods:**

For this cross-sectional clinical method applicability study, capillary and venous blood was collected in duplicate and analyzed at local primary health care centers. For a heterogeneity test, the tests were performed by an expert, and by a team of local nurses. The study was conducted in a multicenter design in rural and urban Aceh, Indonesia in 2019, and included a total of 533 adults. We mainly used Bland-Altman plots to assess the number of readings within the 95%-limits of agreement (LoA) and Deming regressions.

**Results:**

The results show a mean difference between capillary HbA1c on the test device and the reference method of −0.54 [CI_0.95_ = −1.6933; 0.6048] with 5.21% of measurements outside the LoA and a Pearson’s *r* = 0.91 in the Deming Regression. There is no significant difference in test concordance between local nurses and the expert (4.23% versus 5.13% results outside the LoA [CI_0.95_ = −0.0331; 0.0511]).

**Conclusions:**

TSB POC for analysis of HbA1c is an acceptable alternative for accessible monitoring of diabetes patients under these conditions. This method could provide access to high-quality diagnostic decisions through regular and cost-effective HbA1c monitoring directly in healthcare facilities, thus providing better access to essential health services.

## Plain language summary

People with diabetes in low- and middle-income countries often lack access to reliable and affordable tests to monitor their long-term blood sugar levels, known as HbA1c. This study tested a simple tablet- or smartphone-based device to measure HbA1c directly at primary health centers. The study was conducted in urban and rural areas of Indonesia with 533 participants. The results showed that the device’s measurements were almost identical to laboratory tests. There was hardly any difference in the results, regardless of whether the tests were carried out by experienced experts or by nurses in the field. This method could provide a basis for regular and affordable HbA1c testing to improve diabetes care and access to basic health services for patients in these regions.

## Abstract (web summary)

Rhode et al. investigate the applicability of smartphone-based point-of-care diagnostics for monitoring HbA1c levels of diabetes patients under real-world conditions in a lower-middle income setting. They discover that the method offers an acceptable alternative for accessible monitoring under these conditions.

## Introduction

Diabetes causes significant morbidity and mortality worldwide^1–3^. As of 2019, four out of five people with diabetes lived in low- and middle-income countries (LMICs), and this share continues to increase^4^. In Indonesia alone, the number of adults with diabetes was estimated at 19.5 million in 2021^5^. However, evidence has shown that despite its growing burden only 40% of people with diabetes are diagnosed and 20% achieve control of their disease^6,7^. Finally, the cost of diabetes accounts for $ 1.3 trillion, or 1.8% of global GDP^8–11^.

One critical challenge to improving diabetes care in LMIC settings is the lack of accurate, affordable, mobile monitoring of HbA1c at point-of-care (POC) to facilitate diabetes diagnosis and improve glucose management, especially in rural or geographically isolated communities^11–14^. To date, most mobile POC technologies have suffered from a lack of applicability when used in field-based settings^13^. Recently developed POC diagnostics can facilitate these analyses via smartphone or tablet attachments. These tablet- and smartphone-based (TSB) devices, such as the AINA Blood Monitoring System, have the potential to overcome this poor applicability by minimizing both, application-driven and human errors and facilitating easy, automatic transfer and storage of blood test results in the application’s (app’s) physician dashboard^15^. Additionally, the use of these devices may offer a cost-effective alternative compared with the current reference standard in a local professional laboratory, providing an approach to improve accessibility to HbA1c monitoring, especially in LMICs. Although simple POC blood glucose measurements are practical and already widely available in many parts of the world, access to the determination of HbA1c levels, which can be more meaningful and recommended for the long-term monitoring of diabetes, is still limited^16^.

In this study, we sought to test the applicability of a TSB POC device by validating it against a local laboratory-based measure of glycated hemoglobin (HbA1c) for the monitoring of diabetes in a resource-constrained, real-world setting of rural Indonesia in Aceh. The Human Development Index (HDI) of Aceh province was 0.719 in 2019, which is similar to the Indonesian average^17^. The burden of diabetes in this region has increased over the last decades, as evidenced by an increase in disability-adjusted life years (DALYs), between 2006 and 2016 from 2.16 to 3.35 million (+55 %)^18^. A recently published study examining access to healthcare services for people with diabetes during the COVID-19 pandemic showed that only 9.5 % of patients had access to blood glucose monitoring, 30.1 % of patients attended diabetes consultations, and the diabetes complication rate was high at 24.6 %^19^. Our study results show that the measurements of the TSB POC device are almost identical to the laboratory tests and that there are hardly any differences in the results, regardless of whether the tests are carried out by experienced experts or by nurses on site. Thus, the TSB POC method for HbA1c analysis under these conditions is an acceptable alternative for accessible monitoring of diabetes patients and could provide a basis for regular and affordable HbA1c testing to improve diabetes care and access to basic health services for patients in these regions.

## Methods

### Study steps

First, we tested the general concordance of the TSB POC device by comparing the analysis results as measured directly in the primary health care center to the results from the local laboratory. Next, we performed a heterogeneity test in which we had local nurses perform the TSB POC analysis in the same setting to determine real-world differences in test performance between local nurses and TSB POC experts. Finally, we tested the TSB POC method by comparing capillary and venous biomarker outcomes to determine if there were differences in comparability by blood type.

### Data source

The study took place in two districts of the province of Aceh in northwestern Indonesia, more specifically the provincial capital Banda Aceh, and its surrounding peri-urban district Aceh Besar (Supplementary Fig. II). This setting allowed us to test the device’s performance in a middle-income setting under tropical climate conditions^20,21^.

Participants in this study were sampled from among those individuals who were concurrently enrolled in the study “Using peer education to improve diabetes management and outcomes in a low-income setting: a randomized controlled trial (Aceh, Indonesia)” (ISRCTN registration number: ISRCTN68253014; www.isrctn.com)^22^. This randomized controlled trial (RCT) investigates the effect of peer education sessions for type 2 diabetes on diabetes-related outcomes in the province of Aceh within local primary health care posts, called “Puskesmas”. The inclusion criteria for the parent RCT were as follows: 1. treated for type 2 diabetes in the respective Puskesmas in the district of Banda Aceh or Aceh Besar, 2. not enrolled in any other study, 3. agreed to undergo the whole process of data collection and blood analyses, 4. agreed to carry out all biological measures included in the protocol, and 5. were between 20 and 89 years old. All patients who were pregnant or unable to attend peer education sessions were excluded from the study. Participants for the RCT were identified and recruited through patient lists at Puskesmas. All patients from these lists who met the above-mentioned inclusion criteria were contacted by Puskesmas staff and asked if they would like to participate in the study. Puskesmas that refused to participate or were very remote (>30 min by car from the local reference laboratory) were excluded. A total of 533 participants from 31 Puskesmas who were also willing to participate in our validation study were included in the study. The detailed power and sample size calculations are provided in Supplementary Methods IV, V. To ensure high quality, blood collection requirements have also been standardized based on the “WHO guidelines on drawing blood”^23^.

The data for this multi-centered method applicability study was collected between March 01, 2019 and April 09, 2019 (Supplementary Fig. I).

**Fig. 1:**
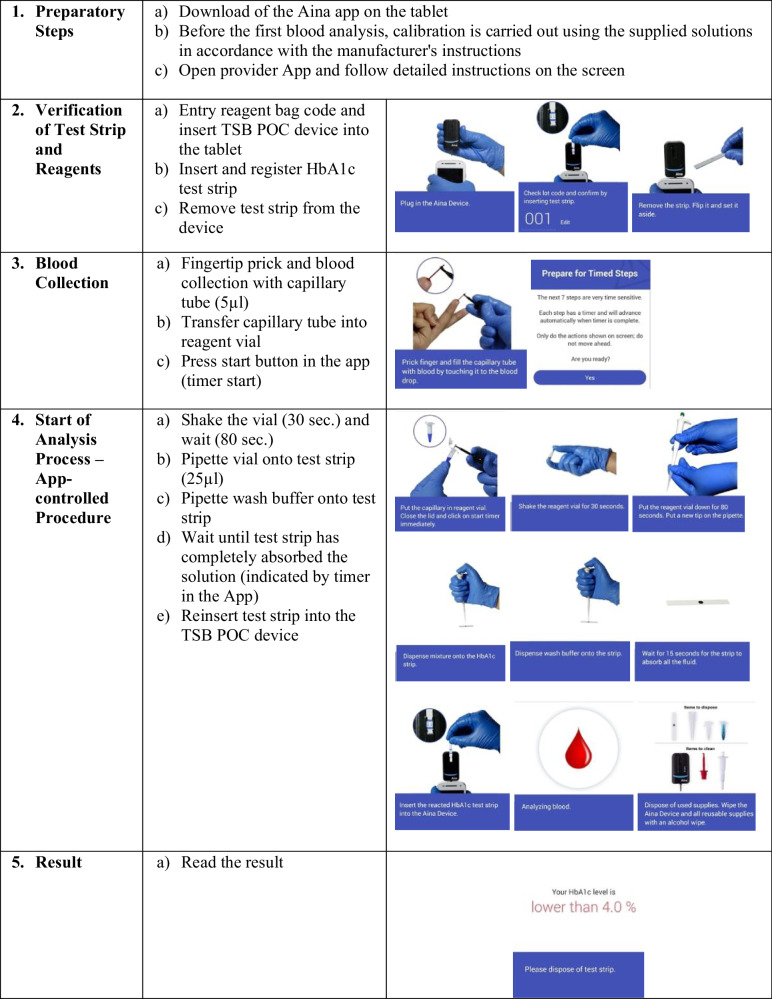
TSB POC Test Steps. TSB POC Device Test Principle in detailed steps using the Boronate affinity method.

The study was performed with the already commercially available and “Conformité Européenne” (CE) certified “AINA Blood Monitoring System” from the company “Jana Care”^24^. The associated and used app of the manufacturer “JanaCare” is called “Aina Station App”. This TSB POC technology analyzes the blood values using a blood volume of 5 μL whole blood (capillary or venous) via the boronate affinity method within 3 minutes. Adding blood to the reagent causes lysis of the erythrocytes and precipitation of all the hemoglobin and the boronic acid conjugates bind to the glycosylated hemoglobin. After an aliquot of the reaction mixture is applied to the test strip, all the precipitated hemoglobin, conjugate-bound and unbound, remains on top of the filter. The unbound boronate is removed by adding the wash buffer. Contents of the HbA1c test kit were test strips, reagents (lysing agent and a blue boronic acid conjugate), wash buffers, capillary tubes for sample collection, and pipette tips. The exact analysis steps are shown in Fig. 1. Further clinical-chemical methodological details of HbA1c measurement with the TSB POC device can be obtained from the “Analytical Performance Summary” provided by the manufacturer^15,24^.

### Collection of participants’ characteristics

As part of the baseline data collection for the RCT, demographic data and additional biomarkers of the study participants were collected using a questionnaire and supplementary blood value analyses. From this, we used information on the diagnosis of type 2 diabetes, and biomarker levels of hemoglobin, HDL, total cholesterol, and triglycerides to characterize our study samples (Table 1). The definition of hypertriglyceridemia and hypercholesterolemia was set according to current recommendations with triglyceride levels above 1.7 mmol/l and total cholesterol levels above 5.2 mmol/l^25,26^. All participants had given their informed consent to participate in the method at the beginning of the study before the data was collected.

**Table 1:** Baseline Characteristics

		Total Observations (*n* (%))
**Having Diabetes** (%)	100	477 (100%)
**HbA1c** (mmol/mol, mean, (range))	84.5 (28–166)	477 (100%)
SD	26.03	
**Hemoglobin** (g/dl, mean, (range))	13.7 (10–20)	477 (100%)
SD	1.63	
**HDL** (mmol/l, median)	1.13	476 (99.8%)
IQR	0.98–1.35	
**Total Cholesterol** (mmol/l, median)	5.50	476 (99.8%)
IQR	4.7–6.4	
**Triglycerides** (mmol/l, median)	1.37	476 (99.8%)
IQR	1.04–1.94	

Characteristics of the study participants in the primary health care centers. Having diabetes is defined as meeting one of the following criteria: HbA1c ≥ 6-5 mmol/mol, diagnosed by a physician or taking antidiabetic medication or insulin. *HbA1c* glycated hemoglobin, *HDL* high density lipoprotein. *n* (%): number of available data/observations from the total number of inclusion samples in percent.

### Collection of Laboratory HbA1c Data

**Fig. 2:**
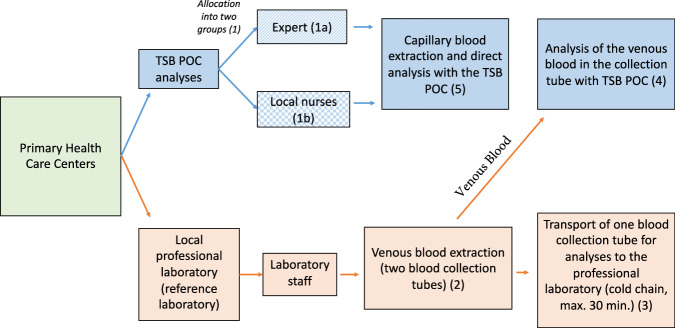
Study procedure. All blood collections originated at the 31 different primary healthcare centers (highlighted in green). The analysis steps undertaken for the evaluation with the TSB POC method are shown in blue. The path taken by the blood samples for analysis with the local laboratory is shown in red. TSB POC: tablet- and smartphone-based point-of-care.

The data collection process is depicted in Fig. 2. First, primary health centers were divided into two groups (1). In one group, an expert performed the TSB POC analyses (1a). An expert was defined as a person who had the following characteristics: 1. medical background, 2. direct intensive one-to-one training by Jana Care staff, and 3. previous experience using TSB POC devices. This individual performed the first 277 measurements to determine if the TSB POC device generally worked in this setting. In the other group, TSB POC analyses were performed by five different local field workers with nursing background (1b) - these were the final 206 measurements, based on the split by Puskesmas. The local nurses had no experience in using TSB POC devices and were previously trained by our research team for a total of nine hours throughout four days on the standard operating procedures for the study and in the use of the TSB POC device. Guidelines and training materials were provided by “JanaCare”. The devices were calibrated and tested before use according to the manufacturer’s operating instructions^24^.

In parallel, venous whole blood was drawn from the patients in duplicate by health professionals from the local professional laboratory (2). For this purpose, two 3 ml EDTA K2 blood collection tubes were drawn from the participants. One of the blood collection tubes was immediately sent to a well-established local clinical laboratory, which served as a reference sample (3). The transportation time was at a maximum 30 minutes under cold chain (+20 °C to +24 °C). The second tube was directly analyzed with the TSB POC tests in the health care center (4). Immediately, within 30 minutes, after venous blood collection, according to the previous grouping, either the expert or one of the five trained local nurses collected a few drops of capillary blood from the patient with a finger stick to analyze the HbA1c level with the TSB POC instruments (5).

As a reference method and local reference standard, HbA1c was analyzed with the BIO-RAD D-10, a national glycohemoglobin standardization program-certified method, using venous whole blood in the professional laboratory^27–29^. The level of the biomarker was determined by using the International Federation for Clinical Chemistry (IFCC) method and the results are accordingly in the unit mmol/mol^30–32^. The TSB POC method was applied using either 5 μL fresh venous blood in EDTA-K2 blood collection tubes (HbA1c, venous) or 5 μL fresh capillary blood from a finger stick (HbA1c, capillary).

For data collection purposes, the Aina Station App, developed for the use of the Aina Blood Monitoring System from the manufacturer “Jana Care”, generated a patient identification number for every sample and transferred the TSB POC blood results via Wi-Fi to a pre-generated physician dashboard account. The reference standard test results of comparator results from BIO-RAD D-10 were provided by the local laboratory on printed laboratory results sheets for each participant.

### Statistics and reproducibility

The program STATA version 15 (StataCorp LLC, Texas, USA) and Rscript (R) version 4.3.2 (2023-10-31; The R Foundation) was used for all analyses.

In all analyses, only study subjects with available hemoglobin (Hb) values were included. Blood samples with Hb values outside the specified working range of the TSB POC device of 10-20 g/dl were excluded to prevent false high or false low measurements. Due to this, 56 samples (10.5%) were excluded (Supplementary Methods I). Study participants with missing data on any of the items were excluded from data analysis, as well.

Blood samples outside the analytical measuring range (AMR, the range that the device can measure) of the TSB POC instruments of 20 - 140 mmol/mol were excluded. This affected three samples (0.6 % of test results) in our study (Supplementary Method VI). Furthermore, we excluded 62 of the total 677 (431 capillary TSB POC measurements + 246 venous TSB POC measurements) analyses performed due to application and analysis errors during the test run on the TSB POC device (9.0 % of test results) (Supplementary Method II, III).

Descriptive characteristics of the study population were analyzed for normally distributed continuous variables as means ± standard deviations (SD), for non-normally distributed variables as ranges or medians and interquartile ranges (IQR), and for categorical variables as counts and proportions. To assess normality Shapiro-Wilk tests were performed.

**Fig. 3:**
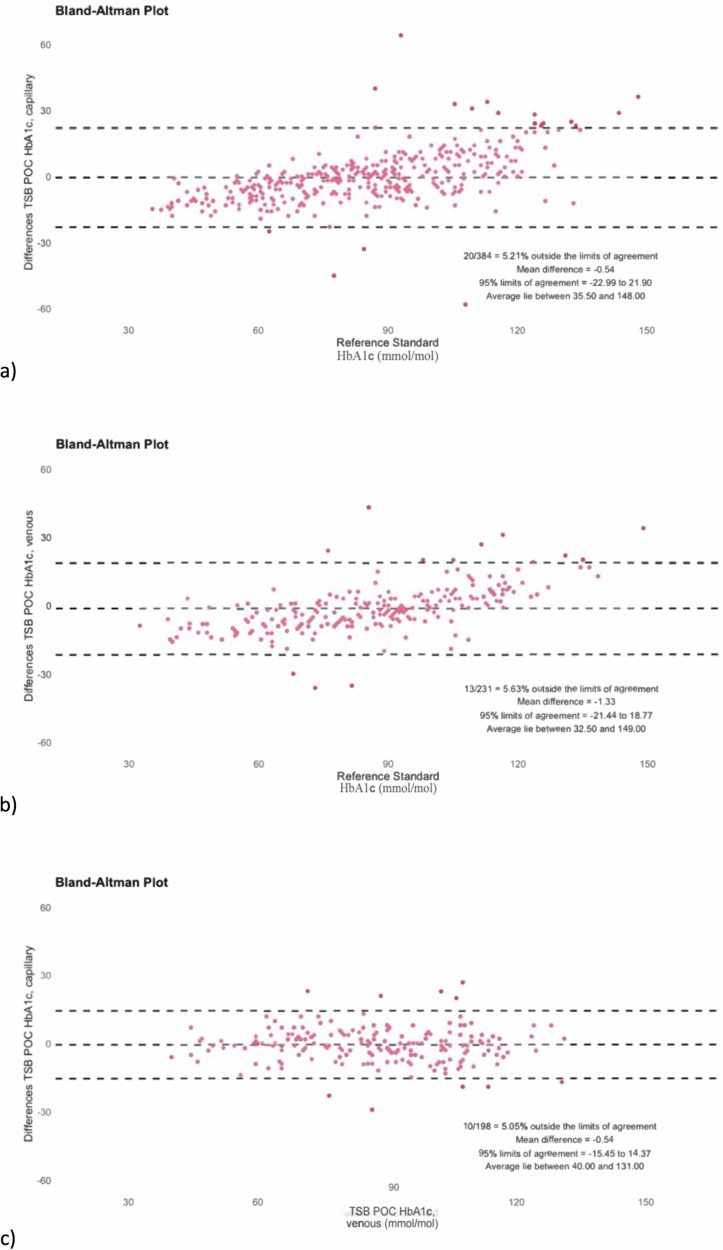
Bland-Altman analyses for HbA1c measurements with different blood types. Shown in each graph is the TSB POC method using capillary blood (**a**) or venous blood (**b**) compared to the reference standard. The performance of the TSB POC method when comparing venous and capillary blood samples on the TSB POC is shown in (**c**). Accordingly, in (**c**) the TSB POC method with venous blood represents the reference. Grey zone: 95% limit of agreement area.

First, we conducted a Bland-Altman analyses to compare the pairs of HbA1c readings (TSB POC versus local laboratory) and calculated the bias as well as the number of measurements outside the limits of agreement (LoA)^33,34^. The bias was set as the mean difference between the two measurements (reference method-TSB POC). The LoA was then determined as bias ± 1.96 x SD^35^. The analytical results over the entire range of measured HbA1c values were plotted graphically in Bland-Altman diagrams to illustrate the agreement between the methods (Figs. 3 and 5). Here, the difference between the TSB POC values and the reference method (y-axis) was plotted against the values of the reference method (x-axis).

For the additional clinical assessment of comparability by the in-range method, clinically acceptable limits of ± 10%, 15%, and 20% deviation from the respective reference method (limit of agreement) were established (Supplementary Table II)^36–38^.

The statistical significance of the differences between the HbA1c values of the respective comparison pairs was tested using a paired *t*-test with a significance level of 0.05.

For the analysis, three different comparison models were performed. First, capillary whole blood was analyzed on the TSB POC devices and compared to the local laboratory Secondly, the same test was repeated with venous whole blood to investigate possible differences between the use of venous and capillary blood on the TSB POC device caused by different blood properties. Finally, we performed a direct comparison of the two blood samples, capillary and venous, on the TSB POC device to increase confidence that potential discrepancies in test results were not due to the use of different sample matrices when using the reference method^39–41^.

**Fig. 4:**
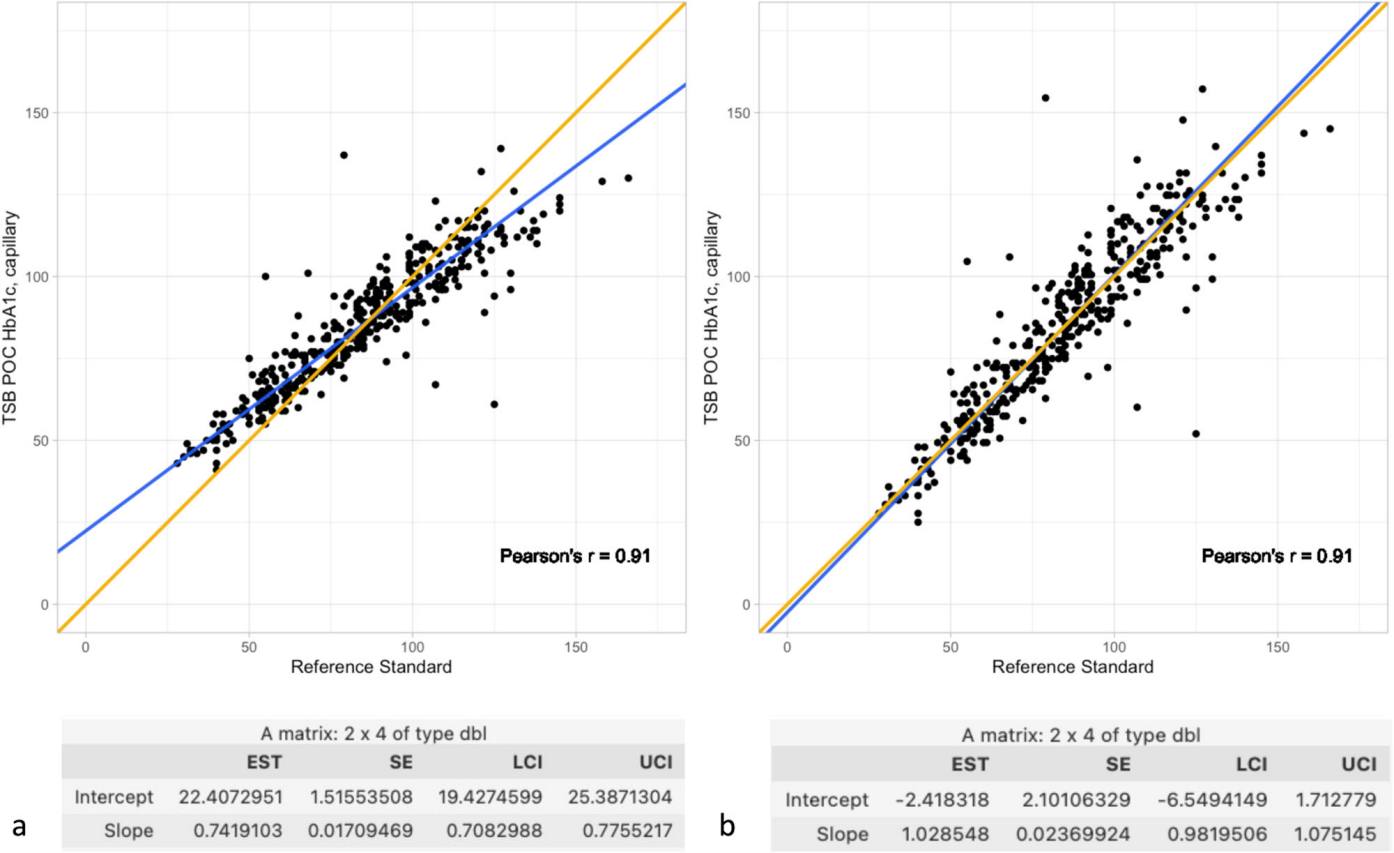
Deming regression. The Deming regression analysis shows excellent agreement with a Pearson’s r of 0.91 when comparing capillary blood using the TSB POC method and the reference standard. **a** shows the original Deming regression analysis. **b** presents the results of the same Deming regression analysis after taking into account a correction factor according to the formula: (TSB POC capillary values - original intercept) * 1/ original slope. EST estimated value, SE standard error, LCI lower confidence interval, UCI upper confidence interval. *n*-value for both analyses: 384 (in accordance with valid capillary TSB POC measurements (see Supplementary Methods II)).

Second, we performed a Deming regression analysis with calculation of Pearson’s *r* and calculated the bias at different HbA1c concentrations to get an insight into whether the two methods give statistically and clinically the same results (Fig. 4a). In a subsequent step, we corrected the values of the Deming regression with a correction factor corresponding to the reciprocal of the intercept and slope values of the previous Deming regression (Fig. 4b), to account for a possible general deviation factor caused by, for example, the use of different analyzers. For this analysis method, capillary blood was tested on the TSB POC device against the reference standard.

Formula for the calculation of the correction factor: (TSB POC capillary values - original intercept) * 1/ original slope

Finally, in order to investigate whether the TSB POC method can be performed by local nurses and present a viable alternative for monitoring patients with diabetes in LMICs, we conducted a heterogeneity check with five local nurses. For this purpose, capillary blood on the TSB POC device was tested against the reference method. We decided not to use venous blood for this method because this study aims to investigate the suitability of these low-cost and easy-to-handle point-of-care diagnostics under local conditions. Since the potential use of these devices in local primary health care centers will be done with the less invasive finger stick using capillary blood this method depicts the realistic setting.

### Reporting summary

Further information on research design is available in the Nature Portfolio Reporting Summary linked to this article.

## Results

### Characteristics of the Samples

In Table 1 we summarize the characteristics of the sample population and the blood samples.

The mean for HbA1c levels was shown to be 84.4 mmol/mol with a standard deviation of 26.1 mmol/mol. We also found moderate levels of hypercholesterinemia (median total cholesterol of 5.6 mmol/l and a median high-density lipoprotein (HDL) of 1.13 mmol/l) in the overall sample. Hypertriglyceridemia (triglyceride levels > 1.7 mmol/l) could not be detected in the study sample^25^. The median of triglyceride values was 1.38 mmol/l.

Table 1 shows the wide range of both hemoglobin (6.2-20 g/dL) and HbA1c (27-166 mmol/mol) values among the samples. The reference values in Table 1 correspond to the measurements with the professional local laboratory.

### Main findings

When comparing the measured values of the TSB POC method and the local laboratory-based reference standard, approximately 85 % of the measured values (TSB POC capillary: 84.51 % and TSB POC venous: 84.78 %) were within the 20 % deviation range and approximately 62 % of the measured values (TSB POC capillary: 60.89 % and TSB POC venous: 63.04 %) were within the 10 % deviation range from the reference method. Testing venous versus capillary blood on the TSB POC device showed 66.67% of the test results were within the 10% deviation range (Supplementary Table II). Respective means were 87.9 [CI_0.95_ = 85.1; 90.7] mmol/mol for HbA1c capillary, 87.4 [CI_0.95_ = 84.6; 90.1] mmol/mol for HbA1c venous, and 86.1 [CI_0.95_ = 82.5; 89.7] mmol/mol for the reference standard. Supplementary Fig. III shows the corresponding median HbA1c measurements for the different methods, while Supplementary Table I shows the descriptive statistics of the different analysis methods.

TSB POC venous and TSB POC capillary blood test results were comparable to the results from the reference laboratory. In another separate analysis comparing the use of capillary and venous blood, we found that the use of capillary blood on the TSB POC device performed best with the reference method (Fig. 3 a). Here, the Bland-Altman-Analysis showed a mean difference in HbA1c of -0.54 mmol/mol with 5.21% of measurements outside the LoA (95% limits of agreement: -23.0 and 21.9 mmol/mol). In comparison, the test performance of venous blood on the TSB POC device against the reference method (Fig. 3 b) showed slightly higher non-significant deviations (mean difference in HbA1c: -1.33, 5.63% outside the 95% limits of agreement of -21.4 and 18.8 mmol/mol). With only minor, not significant deviations in the mean differences between the limits of agreement when comparing the use of capillary and venous whole blood for the TSB POC method, the TSB POC method can analyze venous blood samples as accurately as capillary blood samples [CI_0.95_ = −0.0329; 0.0413].

We found that the overall mean differences between venous and capillary whole blood analysis using the TSB POC method (−0.540) and the TSB POC capillary blood method compared with the reference method (-0.544) were almost identical (Fig. 3c).

The Deming regression showed a slope of 0.74 [CI_0.95_ = 0.71; 0.78] and an intercept of 22.41 [CI_0.95_ = 19.43; 25.39] (Fig. 4a). Despite this fact, a high Pearson’s *r* = 0.91 was found. By applying the correction factor calculated from the slope and intercept deviations, these values could be adjusted to a highly acceptable intercept of −2.42 [CI_0.95_ = −6.55; 1.71] and a slope of 1.03 [CI_0.95_ = 0.98; 1.08] (Fig. 4b).

**Fig. 5:**
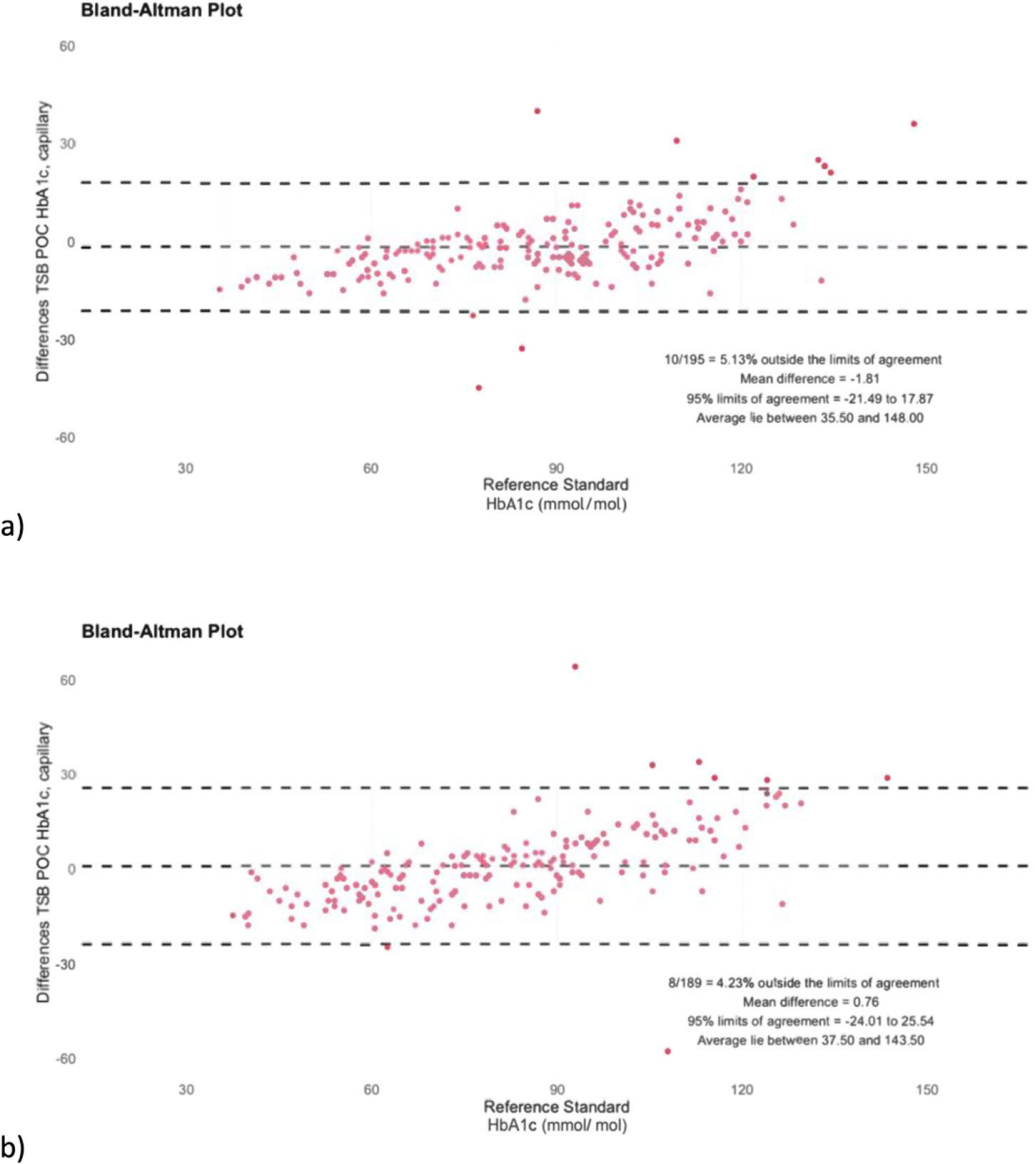
Heterogeneity check of the TSB POC method. The TSB POC method heterogeneity check by comparing results of the TSB POC expert (**a**) with those of the different nurses (**b**).

As Fig. 5 shows, the heterogeneity check of testing the use of capillary blood on the TSB POC device compared to the reference method with nurses instead of the expert provided similar test results. The HbA1c analyses with the TSB POC device had a mean difference of 0.76 and 4.23% of results outside the limits of agreement. The test performance of the nurses was comparable to that of the expert, who showed a mean difference of -1.81 and 5.13% of results outside the limits of agreement [CI_0.95_ = −0.0331; 0.0511].

This finding coincides with the results of error calculations we performed. For the nurses, 92.0% of TSB POC tests were performed correctly without an error shown on the device. This indicates a similar performance to that of the expert, whose rate was 90.5% [CI_0.95_ = −0.0296; 0.0596] (Supplementary Method III).

Missing calculations regarding general TSB POC test feasibility and error testing show good performance of the TSB POC test also under local non-laboratory conditions, with 89.3% of the capillary blood tests and 93.9% of venous blood tests performed correctly (see Supplementary Methods II,III).

## Discussion

In this study, we found high comparability and applicability of a smartphone- and tablet-based point-of-care (TSB POC) method for analyzing HbA1c levels, allowing this technology to be used to monitor patients with diabetes under real-world conditions in a resource-limited setting. Our study shows that the TSB POC method performs on par with an on-site professional laboratory and is a viable, affordable alternative for monitoring people with diabetes and cardiovascular risk in resource-limited settings. In addition to the measurement accuracy of the device, we have also demonstrated the applicability of the method under local conditions in this study. Previous studies have mainly focused on optimal conditions in laboratories, although it is well known that there is a large gap between the increasing prevalence of patients with poorly controlled diabetes and appropriate monitoring approaches to prevent serious diabetes-related complications in these patients, particularly in LMIC settings. The present study represents an important departure from this literature by evaluating point-of-care technology directly in local primary care centers. To date, many POC technologies have suffered from a lack of reliability when deployed in the field, while others have never been systematically tested in field conditions.

To our knowledge, our study is the first comprehensive transfer study to evaluate the applicability of a novel and easy-to-implement TSB POC method for monitoring HbA1c levels under real-life, representative conditions directly in primary care centers in a resource-limited setting compared with the local laboratory, including use by local nurses.^11,12,37,42^.

Overall, the results of both the Bland-Altman analyses and the Deming regression, with a Pearson’s r of 0.91, show high agreement between the TSB POC method using capillary blood and the reference method. Although we were initially able to find a statistically significant indication of a proportional and constant difference between the two methods in the Deming regression with deviating slope and axis values (Fig. 4a). However, the high Pearson’s r indicates, that the TSB POC device’s measurement error is a systematic one and could be corrected by inversely applying the slope and intercept factors. By applying this correction factor, it was possible to adjust these values so that there was no longer a statistically significant difference between the two methods across all measured values, both proportional and constant (Fig. 4b). Using this correction factor, the results showed excellent agreement between the values, both for low and high HbA1c values, assuming stability between the different lots of reagents.

As different literature sources describe a general deviation of HbA1c analysis results when using different analyzers, we also asked whether the general application of different HbA1c analysis methods had any relevance in assessing our results^39,41^. In contrast to what has been suggested in the past, we found in our analyses that there was an acceptable difference in agreement when using capillary or venous blood. Previous studies have suggested that correlations between blood from the same collection types were highest^43,44^. There was also no statistically significant difference between measurements done by trained experts and local nurses.

These findings are important for three reasons: First, they show that the use of blood with different properties - venous blood in the professional reference laboratory and capillary blood on the TSB POC devices - had no influence on the evaluability of the analysis results. Thus, the results are unlikely to be affected by the type of blood sample. Second, based on these results, the TSB POC method can also be used with equal consistency with the less invasive and more applicable method of capillary blood collection, eliminating the need for the more complex venous blood collection. Finally, we found that the tests performed equally well when applied by local nurses or by TSB POC experts, underlining the potential for TSB POC to be used in local primary health care centers and for field research purposes without compromising the test performance compared to the reference method.

The result of the in-range method must also be considered. The result that on average about 62% of the test results were within the 10% deviation range is acceptable, but could certainly be improved. Looking at the comparative values of capillary and venous blood, both tested using the TSB POC method, these achieve, around 67% of the values within the 10% deviation, a higher value than the comparison between TSB POC and the reference method. This also allows the conclusion to be drawn that the use of different analysis methods or devices themselves may have contributed to deviations in the test results^45,46^. Possible inter-device deviations have already been described in various previous studies.

This study also has some limitations: First, we tested the device on a group of people with diabetes with high HbA1c levels, and for ethical reasons with only a single capillary blood sample. Therefore, receiver operating characteristic (ROC) analysis, rule-in versus rule-out analysis or determination of positive and negative predictive values, as well as reproducibility tests for the determination of coefficients of variation were not performed.

Second, we conducted the study in a middle-income setting where basic training for nurses is already well-established. For this reason, the conclusions of the heterogeneity tests are limited to the use of the test by nurses with this background and cannot automatically be transferred to a setting with health workers without basic nursing training. Finally, in our setting, we had only one local reference standard available. A validation against different reference methods would offer the possibility to confirm the performance of the reference methods themselves and to investigate the influence of long transport times of the blood collection tubes at high temperatures on the test results, as is the case when local POC methods are unavailable. We aimed to address this issue by selecting the best possible local reference laboratory. Third, above the concentration of 75 mmol/mol, we found few results with significant differences. Possible causes that may be major contributors to these discrepancies between platforms include hemoglobinopathies. Even though we were able to identify severe anemias by determining hemoglobin levels and exclude them from the analyses, it cannot be ruled out that other hemoglobinopathies that are associated with no changes in hemoglobin levels may have contributed to these discrepancies^41,47,48^.

In addition to these limitations of the study, there are also some limitations of the TSB POC devices themselves, which include the following: First, the analytical measurement range of the TSB POC method is smaller compared to the local professional laboratory. Second, the performance of the TSB POC method must be exact and individuals must be trained in advance. This could substantially reduce higher test error rates during analysis. However, compared to the several years of training of laboratory assistants required to operate the analytical equipment in the local reference laboratory, the TSB POC method, which could be performed under only nine hours of training by the local nurses, is much faster to learn. Even though only correctly performed blood sampling was observed in our observations, other pre-analytical mistakes e.g., insufficient blood sampling (e.g., strong pressure exerted during capillary sampling) or insufficient mixing, can also lead to incorrect analyses. This point should also be addressed by appropriate training. Third, the test reagents must be stored between 2 to 8 °C, which requires the availability of a refrigerator in the testing environment. Nevertheless, the test performance under local conditions also during the heterogeneity check was highly acceptable in our study. Finally, another minor limitation in terms of practical use is that an internet connection is required for the final data transfer of the TSBPOC. However, as the tests themselves do not require an internet connection and the data can initially be stored locally on the respective devices until there is sufficient internet stability for transmission, this limitation was of no major significance in our setting.

The cost of using the TSB POC devices (USD 2.13 per test) was 17.0% of the cost of performing it with the current reference laboratory (USD 12.59/ per test). According to this calculation, the use of TSB POC devices allows to perform 5.9 times as many HbA1c tests to reach the same cost as performing one reference standard test. With an estimated number of 19.5 million adults with diabetes in Indonesia in 2021 receiving Hba1c testing based on current guideline recommendations at least two times per year in case of stable glycemic control or at least quarterly if patients do not meet glycemic goals – the more likely scenario in our setting - this results in an estimated cost saving of USD 204 million per year.

Overall, our findings show the potential to use POC devices in settings where access to laboratories is limited. In settings in which recommended regular HbA1c check-ups^49,50^, cannot be followed, the use of TSB POC devices can be a viable alternative with few drawbacks. Thus, the urgent need for access to better healthcare services for diabetes monitoring outweighs their limitations. This is especially true in rural areas where laboratory monitoring of people with diabetes is not possible. An advantage of the TSB POC method at the level of primary health care centers is the immediate availability of the test results. This allows for immediate feedback to the patient with the possibility of immediate action as well as reducing the risk of loss to follow-up. The interaction between the app and the user makes it possible to reduce analytic errors and facilitate handling. Moreover, the app´s dashboard can help health professionals manage their patients’ disease based on reported HbA1c levels. Most similar POC technologies to date have suffered from a lack of reliability when used in field-based settings or lack of data regarding performance under field conditions^11,13,37,42^.

Taken together, our study shows that the TSB POC method is a non-inferior, applicable, and cost-effective alternative to the current local laboratory-based analytical method for monitoring people with diabetes and cardiovascular risk in low-resource settings. By establishing properly operating and affordable TSB POC-based HbA1c monitoring in primary health care centers, diabetes patients in LMICs with limited laboratory capacity and availability, can be given access to adequate blood analyses and better disease control.

## Supplementary information

Supplementary Information

Description of Additional Supplementary Files

Supplementary Data 1

Supplementary Data 2

Reporting Summary

## Data Availability

All data generated and analyzed in this study are included in this published article and its supplemental information files. The source data used for each individual analysis and the figures and tables generated from them (including the additional figures and tables in the supplementary data file) are in Supplementary Data 1.

## Code availability

The R code used to generate and process the data, as described in the manuscript, is available in Supplementary Data 2.
