# Supplementary material for: Real-world smartphone-based point-of-care diagnostics in primary health care to monitor HbA1c levels in people with diabetes
Source: Commun Med (Lond). 2025 Feb 5;5:37. doi: 10.1038/s43856-025-00743-8 (PMC11799141; doi:10.1038/s43856-025-00743-8)
Supplement: Supplementary file 1 — Supplementary Information [file 43856_2025_743_MOESM1_ESM.pdf]

Supplementary Figure I: Duration and timing of the study

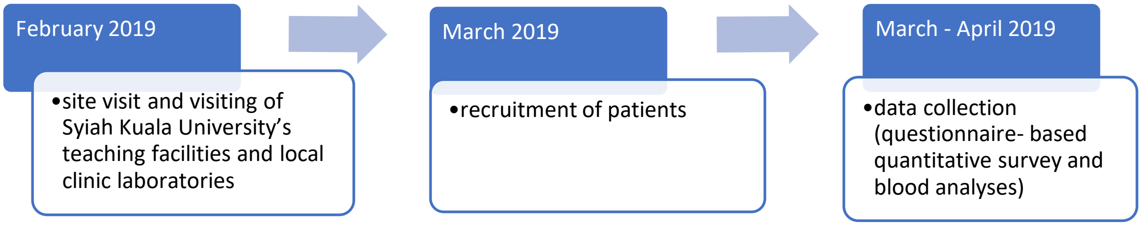

Supplementary Methods I: Process of sample exclusion

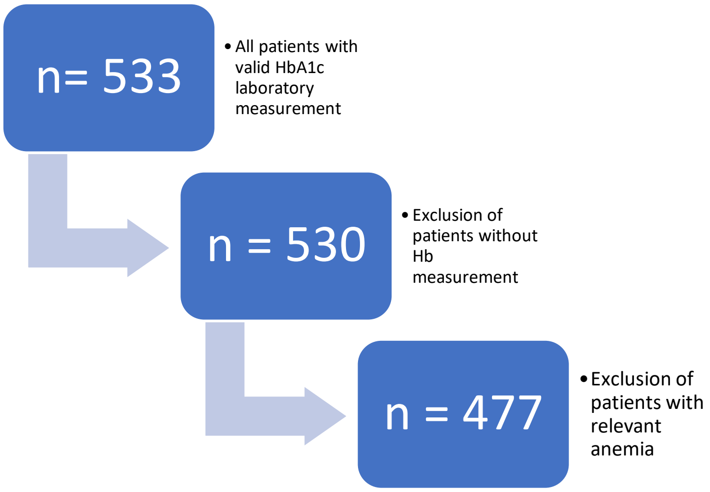

Supplementary Table I: Descriptive Statistics of the Different Analysis Methods

		BIO-RAD D-10			TSB POC Device				
	**n**	**AMR (result range)**	**Result mean**	**(SD)**	**AMR (result range)**	**Result mean**	**(SD)**	**Mean bias**	**% Bias**
**HbA1c,** capillary blood (mmol/mol)	384	18-179 (28-166)	84.71	26.18	20.22-140.44 (41-139)	85.26	19.93	-0.55	**0.65**
**HbA1c,** venous blood (mmol/mol)	231	18-179 (28-166)	85.95	25.80	20.22-140.44 (37-132)	87.29	19.70	-1.34	**1.56**
**HbA1c,** TSB POC comp. (mmol/mol)	198	20.22-140.44 (37-132)	87.35	19.83	20.22-140.44 (41-139)	87.89	20.28	-0.54	**0.62**

Correlation between the TSB POC method and the local reference standard. All analyses - both those performed by the expert and those performed by local nurses - were included. Reference method: HbA1c analyses from venous whole blood with the BIO-RAD D-10. Comparison method: HbA1c analyses each from fresh capillary whole blood or fresh venous blood in EDTA-K2 blood collection tubes with the TSB POC device. For the TSB POC comparison method (TSB POC, comp.), HbA1c analyses from fresh venous blood in EDTA-K2 blood collection tubes were tested against analysis results from fresh capillary whole blood, both analyzed with the TSB POC device.

HbA1c: glycated hemoglobin. TSB POC: tablet- and smartphone-based point-of-care. n: Number of observations. AMR: analytical measurement range – the range that the device can measure. Result Range: range of measured results. SD: standard deviation.

Supplementary Table II: In-Range-Method

Analyte	BIO-RAD D-10	TSB POC Device	n	Limit of Agreement 10%	15%	20%	Mean Div. (%)	(SD)
**HbA1c, total** (mmol/mol)	Local Reference	TSB POC (capillary)	381	**60.89**	**76.90**	**84.51**	**10.97**	0.114
**HbA1c, total** (mmol/mol)	Local Reference	TSB POC (venous)	230	**63.04**	**77.39**	**84.78**	**10.3**	0.108
**HbA1c, total** (mmol/mol)	TSB POC (venous)	TSB POC (capillary)	198	**66.67**	**80.81**	**85.35**	**6.6**	0.064

All analyses - both those performed by the TSB POC expert and those performed by local nurses - were included. The method shows the different observations in percent, which were in a range of 10 %, 15%, and 20 % deviation from the respective reference method (limit of agreement). HbA1c: glycated hemoglobin. n: Number of total observations. Mean Div.: mean divergence between the comparison method and the reference method. SD: standard deviation. Using this method, the performance was best for HbA1c when using venous blood on the TSB POC device (mean divergence: 10.3 %; 77.39 % of tests in the 20 %-limit of agreement). By performing nearly the same test procedure under perfect laboratory conditions the provider of the devices "Jana Care" showed in their "Analytical Performance Summary" [34] that 98.3 % of the capillary blood samples were within 15 % of the reference method for HbA1c.

Supplementary Methods II: Missing calculation due to incorrect analysis on the TSB POC device – total.

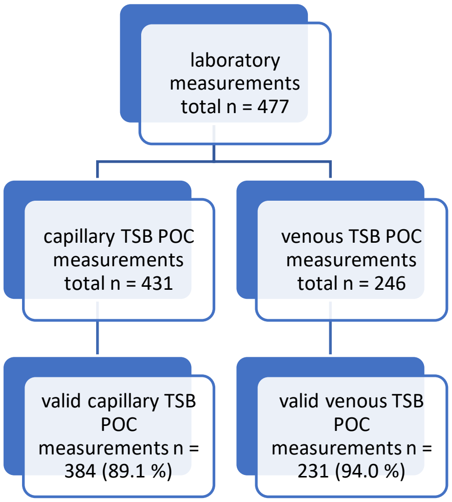

The calculations include all HbA1c analyses that were started on the TSB POC device but not completed due to an error indication in the AINA app. These were triggered by application and analysis errors during the test run. All analyses - both those performed by the expert and those performed by local nurses - were included.

Supplementary Methods III: Missing calculation due to incorrect analysis on TSB POC device - heterogeneity check.

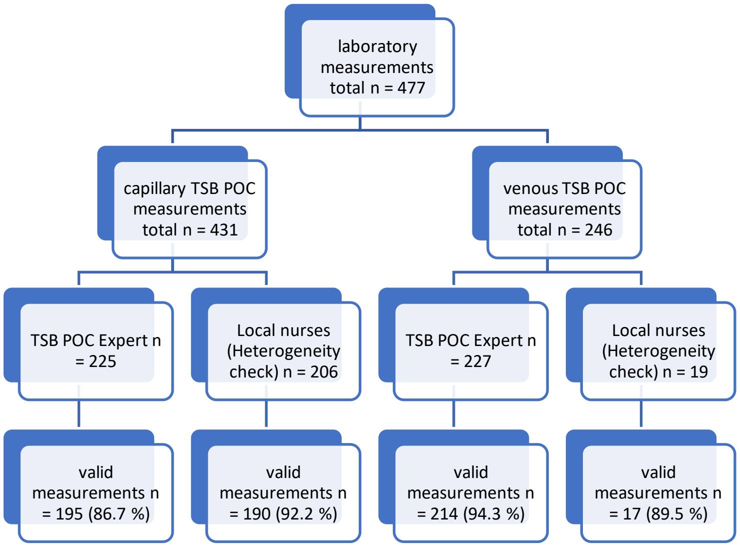

The results shown are subdivided by analyses performed by Expert or local nurses. Calculations include all HbA1c analyses that were started on the TSB POC device but not completed due to an error indication in the AINA app. These were triggered by application and analysis errors during the test run

Supplementary Methods IV: Statistical Analysis Plan.

Concept of statistical test for a confirmatory analysis:
To control for type 1 error, we set the significance level with α_i= 0.01 for each of the five* tested biomarkers (i) to get a significance level of at least α= 0.05 for the overall test where each biomarker contributes 25% to the overall accuracy. H0 will be overruled if the test statistic (T) is in the rejection region Aα. Aα will be defined with P_{H0} (T ∈ Aα) ≤ α. The statistical power (P) is calculated to be at least 80% for each single Biomarker with P_{H0} (T ∈ Aα) = 1-β.
Since the target value is normally distributed, non- inferiority will be proven if H1: μY − μX >- δ, with δ>0 defined as pre-specified margin [−δ, δ] with μY as the expectation of current reference standard measurement, μX as the expectation of TSB POC and δ>0 as margin.
Since the target value is normally distributed, for the equivalence test of the secondary issue, equivalence will be proven if H1: −δ <μY − μX <δ, with δ>0 defined as equivalence range [−δ, δ] with μY as the expectation of current reference standard measurement, μX as the expectation of TSB POC and δ>0 as margin.
The null hypothesis will be tested with a paired sample t-test with:

$$T= \sqrt{\frac{n_x n_y}{n_x+n_y}} \times \frac{\bar{Y}-\bar{X}+\delta}{S}$$

$$t = \frac{\bar{X}_d}{S_{\bar{X}_d}}$$

t=	difference of means/ total variability of samples

Sd=	standard deviation of difference of measurement pairs
Xd=	mean of difference of measurement pairs

μY= mean respective reference standard
μX= mean respective STB RDT

Test results of the t- test will be calculated by using STATA.

** Within the same study setting, we tested 4 additional biomarkers (total cholesterol, high-density lipoprotein, triglycerides, and glucose) in a separate study.*

Supplementary Methods V: Sample Size and Power Calculation of the t-Tests.

- continuous variable; powerandsamplesize.com (Test-1-Mean/ 1-Sample-Non-inferiority or Superiority)

	n	Mean	SD	CRL	MCID	µ	µ0	δ	Power	α
**TC (mg/dl)**	23 (14)	216.3	39.4	242.8	26.5	216.3	216.3	26.5	0.80	1% (5%)
**TAG (mg/dl)**	109 (67)	152.9	79.7	177.1	24.2	152.9	152.9	24.2	0.80	1% (5%)
**HDL (mg/dl)**	65 (40)	48.6	11.9	43.9	4.7	48.6	48.6	4.7	0.80	1% (5%)
**Glucose (mg/dl)**	9 (5)	102.1	26.7	108.3	29.8	102.1	102.1	29.8	0.80	1% (5%)
**HbA1c (%)**	91 (56)	8.5	1.5	8.0	0.5	8.5	8.5	0.5	0.80	1% (5%)

- continuous variable; powerandsamplesize.com (Test-1-Mean/ 1-Sample-Equivalence)

	n	Mean	SD	CRL	MCID	µ	µ0	δ	Power	α
**TC (mg/dl)**	29 (19)	216.3	39.4	242.8	26.5	216.3	216.3	26.5	0.80	1% (5%)
**TAG (mg/dl)**	141 (93)	152.9	79.7	177.1	24.2	152.9	152.9	24.2	0.80	1% (5%)
**HDL (mg/dl)**	84 (55)	48.6	11.9	43.9	4.7	48.6	48.6	4.7	0.80	1% (5%)
**Glucose (mg/dl)**	11 (7)	102.1	26.7	108.3	29.8	102.1	102.1	29.8	0.80	1% (5%)
**HbA1c (%)**	117 (77)	8.5	1.5	8.0	0.5	8.5	8.5	0.5	0.80	1% (5%)

The study comprised patients from 29 teaching health facilities of the Syiah Kuala University settled in Banda Aceh or Aceh Besar. To analyze the overall accuracy of the test. type 1 error rate of 5% is separated into the five different biomarker outcome measurements. whereby each analyzed biomarker is conceded a type 1 error rate of 1%. The highest calculated sample size. which was calculated to be needed for TAG measurement (as the table shows). is used as overall sample size for each biomarker measurement. Using 141 participants for all tests provided us with a minimum 80% power to detect a difference of 24.2 mg/dl in mean TAG using a significance level of p=0.01. We aimed to collect a sample size of 162 participants. which allowed us to lose 21 participants due to drop out or 21 errors in blood analyses.*

* Within the same study setting. we tested 4 additional biomarkers (total cholesterol. high-density lipoprotein. triglycerides. and glucose) in a separate study.

Supplementary Figure II: **Map of the study location in Aceh, Indonesia.**

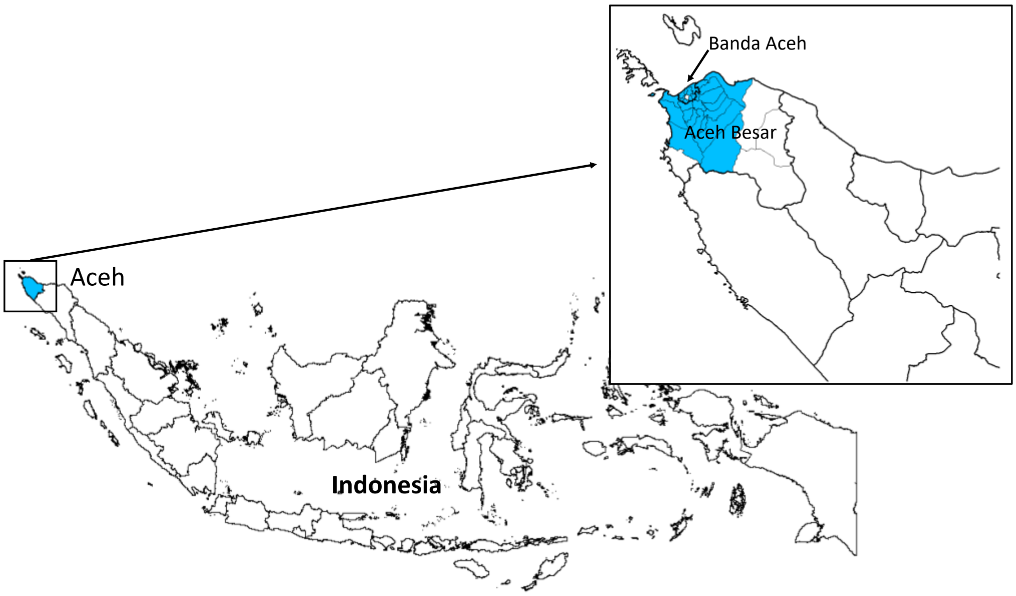

Shown are the Indonesian islands with blue marking of the study location in the northernmost province of Sumatra, Aceh. In the enlargement section, the exact study locations in the rural (Aceh Besar) and urban (Banda Aceh) are also highlighted in blue.

Supplementary Figure III: HbA1c measurements across the different methods.

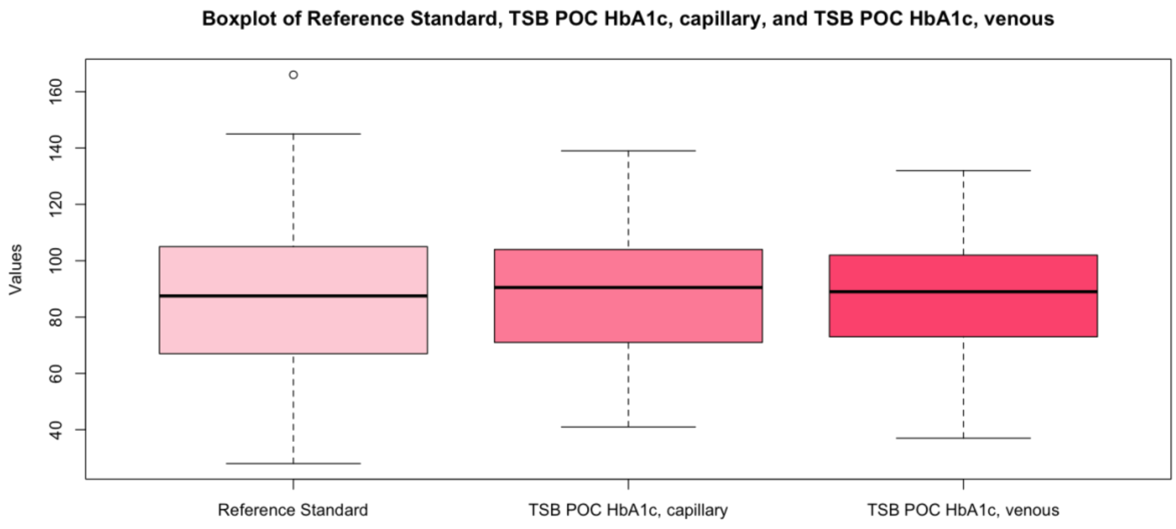

The hemoglobin A1c (HbA1c) values are shown in a boxplot diagram with the respective medians, minima and maxima measured by three different methods. Local reference standard: reference method (bright), TSB POC, capillary: HbA1c from capillary blood analyzed with the TSB POC instrument (medium), and TSB POC, venous: HbA1c from venous blood analyzed with the TSB POC method (dark). Bars: minimum and maximum values. Box: interquartile range, first quartile (25th percentile) to third quartile (75th percentile). In-box-line: median value. Dot: outlier (outside 1.5 times the interquartile range above the upper and below the lower quartile).

Supplementary Methods VI: Missing calculation due to location outside the analytical measurement range of the TSB POC method.

Analyte	n	Missing (n)	Missing (%)
**HbA1c,** capillary	477	3	0.63
**HbA1c,** venous	476	0	0.00

n: Number of total observations. Missing (n): absolute number of counted missing values that were outside the TMR range. Missing (%): percentage of the respective missing values. AMR: analytical measurement range.
