# Supplementary material for: Real-world smartphone-based point-of-care diagnostics in primary health care to monitor HbA1c levels in people with diabetes
Source: Commun Med (Lond). 2025 Feb 5;5:37. doi: 10.1038/s43856-025-00743-8 (PMC11799141; doi:10.1038/s43856-025-00743-8)
Supplement: Supplementary file 2 — Description of Additional Supplementary Files [file 43856_2025_743_MOESM2_ESM.pdf]

Description of Additional Supplementary Files

File name: Supplementary Data 1

File description: All data generated and analyzed in this study are included in this published article and its supplementary information files

File name: Supplementary Data 2

File description: The R code used to generate and process the data, as described in the manuscript.
