# Supplementary material for: Real-world smartphone-based point-of-care diagnostics in primary health care to monitor HbA1c levels in people with diabetes
Source: Commun Med (Lond). 2025 Feb 5;5:37. doi: 10.1038/s43856-025-00743-8 (PMC11799141; doi:10.1038/s43856-025-00743-8)
Supplement: Supplementary file 3 — Supplementary Data 1 [file 43856_2025_743_MOESM3_ESM.pdf]

```r
# Preparation
# Install and load the openxlsx package
if (!require(openxlsx)) {
  install.packages("openxlsx")
}
library(openxlsx)

# Save the selected_data dataframe as an Excel file
write.xlsx(selected_data, "selected_data.xlsx", row.names = FALSE)



# Define filters
reference_samples_ok <- quo(hba1c_p_1 == 1)
tsbpoc_capillary_samples_ok <- quo(hba1c_c_1 == 1 & hba1c_c_2 == 0)
tsbpoc_venous_samples_ok <- quo(hba1c_v_1 == 1 & hba1c_v_2 == 0)
hemoglobin_values_ok <- quo(hb >= 10 & hb_1 == 1)
tsbpoc_capillary_nurses_only_samples_ok <- quo(hba1c_c_1 == 1 & hba1c_c_2 == 0 & HC == 1)
tsbpoc_capillary_expert_only_samples_ok <- quo(hba1c_c_1 == 1 & hba1c_c_2 == 0 & HC == 0)
hdl_ok <- quo(hdl_p_1 == 1)
tc_ok <- quo(tc_p_1 == 1)
tag_ok <- quo(tag_p_1 == 1)


capillary_is_valid_filter <- quo(!!reference_samples_ok & !!tsbpoc_capillary_samples_ok &
!!hemoglobin_values_ok)
venous_is_valid_filter <- quo(!!reference_samples_ok & !!tsbpoc_venous_samples_ok & !!hemoglobin_values_ok)
capillary_nurses_only_is_valid_filter <- quo(!!reference_samples_ok & !!tsbpoc_capillary_nurses_only_samples_ok
& !!hemoglobin_values_ok)
capillary_expert_only_is_valid_filter <- quo(!!reference_samples_ok & !!tsbpoc_capillary_expert_only_samples_ok
& !!hemoglobin_values_ok)


capillary_and_venous_is_valid_filter <- quo(!!capillary_is_valid_filter & !!venous_is_valid_filter)

reference_samples_ok_filter <- quo(!!reference_samples_ok)
hdl_ok_filter <- quo(!!hdl_ok)
tc_ok_filter <- quo(!!tc_ok)
tag_ok_filter <- quo(!!tag_ok)
```

```r
# Apply filters
data_reference_is_valid <- filter(selected_data, !!reference_samples_ok_filter)
data_capillary_is_valid <- filter(selected_data, !!capillary_is_valid_filter)
data_venous_is_valid <- filter(selected_data, !!venous_is_valid_filter)

data_both_is_valid <- filter(selected_data, !!capillary_and_venous_is_valid_filter)
data_venous_and_capillary_is_valid <- filter(selected_data, !!capillary_and_venous_is_valid_filter)

data_capillary_nurses_only_is_valid <- filter(selected_data, !!capillary_nurses_only_is_valid_filter)
data_capillary_expert_only_is_valid <- filter(selected_data, !!capillary_expert_only_is_valid_filter)

data_hdl_is_valid <- filter(selected_data, !!hdl_ok_filter)
data_tc_is_valid <- filter(selected_data, !!tc_ok_filter)
data_tag_is_valid <- filter(selected_data, !!tag_ok_filter)



reference_standard_capillary_values <- data_capillary_is_valid$hba1c_p
reference_standard_venous_values <- data_venous_is_valid$hba1c_p

reference_standard_capillary_and_venous_is_valid_values <- data_venous_and_capillary_is_valid$hba1c_p

reference_standard_nurses_only_capillary_values <- data_capillary_nurses_only_is_valid$hba1c_p
reference_standard_expert_only_capillary_values <- data_capillary_expert_only_is_valid$hba1c_p

tsbpoc_capillary_values <- data_capillary_is_valid$hba1c_c
tsbpoc_venous_values <- data_venous_is_valid$hba1c_v

tsbpoc_both_is_valid_capillary_values <- data_venous_and_capillary_is_valid$hba1c_c
tsbpoc_both_is_valid_venous_values <- data_venous_and_capillary_is_valid$hba1c_v

tsbpoc_capillary_nurses_only_values <- data_capillary_nurses_only_is_valid$hba1c_c
tsbpoc_capillary_expert_only_values <- data_capillary_expert_only_is_valid$hba1c_c



# Baseline Characteristics
mean_hba1c_p <- mean(data_reference_is_valid$hba1c_p)
sd_hba1c_p <- sd(data_reference_is_valid$hba1c_p)

mean_hba1c_p
```

```r
sd_hba1c_p

length(data_reference_is_valid$hba1c_p)
summary(data_reference_is_valid$hba1c_p)


mean_hb <- mean(selected_data$hb)
sd_hb <- sd(selected_data$hb)

mean_hb
sd_hb

length(selected_data$hb)
summary(selected_data$hb)


mean_hdl <- mean(data_hdl_is_valid$hdl_p)
sd_hdl <- sd(data_hdl_is_valid$hdl_p)

mean_hdl
sd_hdl

length(data_hdl_is_valid$hdl_p)
summary(data_hdl_is_valid$hdl_p)


mean_tc <- mean(data_tc_is_valid$tc_p)
sd_tc <- sd(data_tc_is_valid$tc_p)

mean_tc
sd_tc

length(data_tc_is_valid$tc_p)
summary(data_tc_is_valid$tc_p)


mean_tag <- mean(data_tag_is_valid$tag_p)
sd_tag <- sd(data_tag_is_valid$tag_p)

mean_tag
```

```r
sd_tag

length(data_tag_is_valid$tag_p)
summary(data_tag_is_valid$tag_p)



# Fit original deming model
deming_model <- mcreg(reference_standard_capillary_values, tsbpoc_capillary_values,
  error.ratio = 1., method.reg = "Deming",
  method.ci = "analytical", mref.name = "ref.name",
  mtest.name = "test.name", na.rm = TRUE
)

deming_coefficients <- getCoefficients(deming_model)
deming_coefficients

deming_coefficients <- getCoefficients(deming_model)
orig_intercept <- deming_coefficients[1, 1]
orig_slope <- deming_coefficients[2, 1]

plot(deming_model, main = "Deming regression fit")



# Fit corrected deming model
corrected_tsbpoc_capillary_values <- (tsbpoc_capillary_values - orig_intercept) * 1 / orig_slope
corrected_deming_model <- mcreg(reference_standard_capillary_values, corrected_tsbpoc_capillary_values,
  error.ratio = 1., method.reg = "Deming",
  method.ci = "analytical", mref.name = "ref.name",
  mtest.name = "test.name", na.rm = TRUE

corrected_deming_coefficients <- getCoefficients(corrected_deming_model)
corrected_deming_coefficients

plot(corrected_deming_model, main = "Deming regression fit")



# Calculate correlation coefficients
# Calculate Pearson's correlation coefficient
orig_pearson_r <- cor(reference_standard_capillary_values, tsbpoc_capillary_values)
```

```r
corrected_pearson_r <- cor(reference_standard_capillary_values, corrected_tsbpoc_capillary_values)


# Plot
# Set the y-axis limits from 0 to the highest value
options(repr.plot.width = 12, repr.plot.height = 6)

p1 <- ggplot(data = data_capillary_is_valid, aes(x = reference_standard_capillary_values, y =
tsbpoc_capillary_values)) +
  geom_point() +
  geom_abline(
    intercept = getCoefficients(deming_model)[1],
    slope = getCoefficients(deming_model)[2],
    color = "#225cf0ee",
    size = 1.
  ) +
  geom_abline(
    intercept = 0,
    slope = 1,
    color = "#f3b407",
    size = 1.
  ) +
  geom_text(
    x = 150, y = 15,
    label = paste("Pearson's r =", round(orig_pearson_r, 2)),
    color = "black",
    size = 4
  ) +
  labs(x = "Reference Standard", y = "TSB POC HbA1c, capillary") +
  scale_x_continuous(limits = c(0, 175), ) +
  scale_y_continuous(limits = c(0, 175)) +
  theme(text = element_text(size = 16, family = "serif")) +
  theme_light()


p2 <- ggplot(data = data_capillary_is_valid, aes(x = reference_standard_capillary_values, y =
corrected_tsbpoc_capillary_values)) +
  geom_point() +
  geom_abline(
    intercept = getCoefficients(corrected_deming_model)[1],
```

```
    slope = getCoefficients(corrected_deming_model)[2],
    color = "#225cf0ee",
    size = 1.
  ) +
  geom_abline(
    intercept = 0,
    slope = 1,
    color = "#f3b407",
    size = 1.
  ) +
  geom_text(
    x = 150, y = 15,
    label = paste("Pearson's r =", round(corrected_pearson_r, 2)),
    color = "black",
    size = 4
  ) +
  labs(x = "Reference Standard", y = "TSB POC HbA1c, capillary") +
  scale_x_continuous(limits = c(0, 175), ) +
  scale_y_continuous(limits = c(0, 175)) +
  theme(text = element_text(size = 16, family = "serif")) +
  theme_light()

plot(p1 + p2)

dir.create("output", showWarnings = FALSE)
pdf("output/deming_model_plot.pdf")
plot(p1 + p2)
dev.off()




# Bland Altman Plot
create_bland_altman_plot <- function(reference_data, test_data, plot_title) {
  library(ggplot2)
  library(BlandAltmanLeh)

  stats <- bland.altman.stats(
    reference_data,
    test_data,
    two = 1.96,
```

```
    mode = 1,
    conf.int = 0.95
  )
  upper_log <- stats$upper.limit
  lower_log <- stats$lower.limit

  # filter stats$diffs between lower_log and upper_log
  are_outside <- stats$diffs < lower_log | stats$diffs > upper_log
  num_outside <- sum(are_outside)

  bland_altman_text <- sprintf(
    "%d/%d = %.2f%% outside the limits of agreement
    Mean difference = %.2f
    95%% limits of agreement = %.2f to %.2f
    Average lie between %.2f and %.2f",
    num_outside,
    stats$based.on,
    num_outside / stats$based.on * 100,
    stats$mean.diffs,
    stats$lower.limit,
    stats$upper.limit,
    min(stats$means),
    max(stats$means)
  )
# Perform Bland-Altman analysis
  plot_bland_altman <- bland.altman.plot(
    reference_data,
    test_data,
    two = 1.96,
    mode = 1,
    graph.sys = "ggplot2",
    conf.int = 0,
    silent = FALSE,
    sunflower = FALSE,
    geom_count = FALSE,
    xlab = "Reference Standard",
    ylab = plot_title
  )
plot_bland_altman <- plot_bland_altman + theme_minimal() +
    labs(
```

```
    title = "Bland-Altman Plot",
    x = "Reference Standard",
    y = plot_title
  ) +
  theme(
    plot.title = element_text(size = 16, face = "bold"),
    axis.title = element_text(size = 14),
    axis.text = element_text(size = 12),
    panel.grid.major = element_line(color = "#f8f7f7"),
    panel.grid.minor = element_blank(),
    panel.border = element_blank(),
    legend.position = "none"
  ) +
  geom_point(
    color = "#ff004c" # Change the color to red
  ) +
  annotate(
    "text",
    x = 130, y = -45, label = bland_altman_text, size = 4, color = "black"
  ) +
  geom_ribbon(
    aes(ymin = stats$lower.limit, ymax = stats$upper.limit),
    fill = "#f4e9f3", alpha = 0.3
  ) +
  coord_cartesian(ylim = c(-60, 60)) +
  coord_cartesian(xlim = c(20, 160)) +
  scale_x_continuous(breaks = seq(30, 150, by = 30)) +
  scale_y_continuous(breaks = seq(-60, 60, by = 30)) +
  expand_limits(y = c(-60, 60))

 return(plot_bland_altman)
}

create_bland_altman_plot(
 reference_standard_capillary_values,
 tsbpoc_capillary_values,
 "Differences TSB POC HbA1c, capillary"
)

create_bland_altman_plot(
```

```r
  reference_standard_venous_values,
  tsbpoc_venous_values,
  "Differences TSB POC HbA1c, venous"
)
create_bland_altman_plot(
  tsbpoc_both_is_valid_venous_values,
  tsbpoc_both_is_valid_capillary_values,
  "Differences TSB POC HbA1c, capillary"
)

create_bland_altman_plot(
  reference_standard_nurses_only_capillary_values,
  tsbpoc_capillary_nurses_only_values,
  "Differences TSB POC HbA1c, capillary"
)

create_bland_altman_plot(
  reference_standard_expert_only_capillary_values,
  tsbpoc_capillary_expert_only_values,
  "Differences TSB POC HbA1c, capillary"
)



# Box Plot
boxplot(reference_standard_capillary_and_venous_is_valid_values,
  tsbpoc_both_is_valid_capillary_values,
  tsbpoc_both_is_valid_venous_values,
  names = c("Reference Standard", "TSB POC HbA1c, capillary", "TSB POC HbA1c, venous"),
  main = "Boxplot of Reference Standard, TSB POC HbA1c, capillary, and TSB POC HbA1c, venous",
  ylab = "Values",
  col = c("#ff004c37", "#ff004c86", "#ff004cbe")



# Shapiro-Wilk Normality Tests
shapiro.test(reference_standard_capillary_values)

hist(reference_standard_capillary_values)
qqnorm(reference_standard_capillary_values)
qqline(reference_standard_capillary_values)
```

```r
shapiro.test(tsbpoc_capillary_values)

hist(tsbpoc_capillary_values)
qqnorm(tsbpoc_capillary_values)
qqline(tsbpoc_capillary_values)


shapiro.test(tsbpoc_venous_values)

hist(tsbpoc_venous_values)
qqnorm(tsbpoc_venous_values)
qqline(tsbpoc_venous_values)
```
