# Supplementary material for: Real-world smartphone-based point-of-care diagnostics in primary health care to monitor HbA1c levels in people with diabetes
Source: Commun Med (Lond). 2025 Feb 5;5:37. doi: 10.1038/s43856-025-00743-8 (PMC11799141; doi:10.1038/s43856-025-00743-8)
Supplement: Supplementary file 5 — Reporting Summary [file 43856_2025_743_MOESM5_ESM.pdf]

nature portfolio

Corresponding author(s): Sabrina Rhode
Heidelberg University
Heidelberg Institute of Global Health
Im Neuenheimer Feld 130.3
69120 Heidelberg
Germany
sabrina.rhode@uni-heidelberg.de

Last updated by author(s): Dec 7, 2024

Reporting Summary

Nature Portfolio wishes to improve the reproducibility of the work that we publish. This form provides structure for consistency and transparency in reporting. For further information on Nature Portfolio policies, see our Editorial Policies and the Editorial Policy Checklist.

Statistics

For all statistical analyses, confirm that the following items are present in the figure legend, table legend, main text, or Methods section.

n/a	Confirmed	
☐	☒	The exact sample size (*n*) for each experimental group/condition, given as a discrete number and unit of measurement
☐	☒	A statement on whether measurements were taken from distinct samples or whether the same sample was measured repeatedly
☐	☒	The statistical test(s) used AND whether they are one- or two-sided<br>*Only common tests should be described solely by name; describe more complex techniques in the Methods section.*
☐	☒	A description of all covariates tested
☐	☒	A description of any assumptions or corrections, such as tests of normality and adjustment for multiple comparisons
☐	☒	A full description of the statistical parameters including central tendency (e.g. means) or other basic estimates (e.g. regression coefficient) AND variation (e.g. standard deviation) or associated estimates of uncertainty (e.g. confidence intervals)
☐	☒	For null hypothesis testing, the test statistic (e.g. *F*, *t*, *r*) with confidence intervals, effect sizes, degrees of freedom and *P* value noted<br>*Give P values as exact values whenever suitable.*
☒	☐	For Bayesian analysis, information on the choice of priors and Markov chain Monte Carlo settings
☒	☐	For hierarchical and complex designs, identification of the appropriate level for tests and full reporting of outcomes
☒	☐	Estimates of effect sizes (e.g. Cohen's *d*, Pearson's *r*), indicating how they were calculated

Our web collection on statistics for biologists contains articles on many of the points above.

Software and code

Policy information about availability of computer code

Data collection	The open accessable software "ODK" was used for this data collection.
Data analysis	We used the program STATA version 15 (StataCorp LLC, Texas, USA) and Rscript (R) version 4.3.2 (2023-10-31; The R Foundation) for all our data analyses. We have not made use of any other previously published commercial, open source code/ software. We bought a official licence to use the above-mentioned statistiacl software.

For manuscripts utilizing custom algorithms or software that are central to the research but not yet described in published literature, software must be made available to editors and reviewers. We strongly encourage code deposition in a community repository (e.g. GitHub). See the Nature Portfolio guidelines for submitting code & software for further information.

Data

Policy information about availability of data

All manuscripts must include a data availability statement. This statement should provide the following information, where applicable:

- Accession codes, unique identifiers, or web links for publicly available datasets
- A description of any restrictions on data availability
- For clinical datasets or third party data, please ensure that the statement adheres to our policy

Data availability
All data generated and analyzed in this study are included in this published article and its supplementary information files under Supplementary Data 1.

Code availability
The R code used to generate and process the data, as described in the manuscript, is available in the Supplementary Information files under Supplementary Data 2.

Human research participants

Policy information about studies involving human research participants and Sex and Gender in Research.

Reporting on sex and gender	As part of baseline data collection for the RCT "Using peer education to improve diabetes management and outcomes in a low-income setting: a randomized controlled trial (Aceh, Indonesia)" (ISRCTN registration number: ISRCTN68253014; www.isrctn.com), from whose sample the basic data for this substudy were used, demographic data were collected from study participants using a questionnaire. From this, we used information on age, sex, body mass index (BMI), diagnosis of type 2 diabetes, diabetes-associated complications, use of medication for diabetes therapy, presence of hypertension or anemia, and biomarker levels of hemoglobin, HDL, total cholesterol, and triglycerides to characterize our study sample. Diabetes-associated complications included the self-reported presence of retinopathy (vision problems, blindness), kidney problems, wound healing problems, neurological problems (tickling or numbness in the feed or hands, stroke), cardiovascular problems, diabetic foot (ulcer, amputation), and/ or others.<br><br>However, for this substudy submitted to your journal, we did not perform any other sex- or gender-based analyses except for the presentation of the study sample. This is due to the design of the study, which focuses on the validation of the measurement method.
Population characteristics	See above.
Recruitment	Participants in this study were sampled from among those individuals who were concurrently enrolled in the study "Using peer education to improve diabetes management and outcomes in a low-income setting: a randomized controlled trial (Aceh, Indonesia)" (ISRCTN registration number: ISRCTN68253014; www.isrctn.com). This randomized controlled trial (RCT) investigates the effect of peer education sessions for type 2 diabetes on diabetes-related outcomes in the province of Aceh within local primary health care posts, called Puskesmas. The inclusion criteria for the parent RCT are as follows: 1. treated for type 2 diabetes in the respective Puskesmas in the district of Banda Aceh or Aceh Besar, 2. not enrolled in any other study, 3. agreed to undergo the whole process of data collection and blood analyses, 4. agreed to carry out all biological measures included in the protocol, and 5. were between 20 and 89 years old. All patients who were pregnant or unable to attend peer education sessions were excluded from the study. Participants for the RCT were identified and recruited through patient lists at Puskesmas. All patients from these lists who met the above-mentioned inclusion criteria were contacted by Puskesmas staff and asked if they would like to participate in the study. Puskesmas that refused to participate or were very remote (> 30 min by car from the local reference laboratory) were excluded.
Ethics oversight	The study was approved by the ethics committee of the University of Göttingen and the ethics committee of the Syiah Kuala University in Banda Aceh, Indonesia (reference 113000211117) as a sub-study within "Using peer education to improve diabetes management and outcomes in a low-income setting: a randomized controlled trial (Aceh, Indonesia)".

Note that full information on the approval of the study protocol must also be provided in the manuscript.

Field-specific reporting

Please select the one below that is the best fit for your research. If you are not sure, read the appropriate sections before making your selection.

☒ Life sciences ☐ Behavioural & social sciences ☐ Ecological, evolutionary & environmental sciences

For a reference copy of the document with all sections, see nature.com/documents/nr-reporting-summary-flat.pdf

Life sciences study design

All studies must disclose on these points even when the disclosure is negative.

Sample size	A total of 533 participants from 31 community health centers were included in the study.
Data exclusions	The details of the exclusion criteria have been explained in the manuscript. This includes invalide data, study participants who do not fit with the inclusion criteria or were not willing to participate.
Replication	In the manuscript we have described in detailed the procedures of the study so that the study can be replicated by others.
Randomization	The data used for this study was collected during the baseline data collection for an RCT, before any randomization took place. For further details please see the recruitment process mentioned above.
Blinding	The data used for this study was collected during the baseline data collection for an RCT, before any blinding took place. For further details please see the recruitment process mentioned above.

Reporting for specific materials, systems and methods

We require information from authors about some types of materials, experimental systems and methods used in many studies. Here, indicate whether each material, system or method listed is relevant to your study. If you are not sure if a list item applies to your research, read the appropriate section before selecting a response.

Materials & experimental systems

n/a	Involved in the study	
☒	☐	Antibodies
☒	☐	Eukaryotic cell lines
☒	☐	Palaeontology and archaeology
☒	☐	Animals and other organisms
☐	☒	Clinical data
☒	☐	Dual use research of concern

Methods

n/a	Involved in the study	
☒	☐	ChIP-seq
☒	☐	Flow cytometry
☒	☐	MRI-based neuroimaging

Clinical data

Policy information about clinical studies

All manuscripts should comply with the ICMJE guidelines for publication of clinical research and a completed CONSORT checklist must be included with all submissions.

Clinical trial registration	As described in "Recruitment" and in the manuscript, this study was conducted in the framework of the study “Using peer education to improve diabetes management and outcomes in a low-income setting: a randomized controlled trial (Aceh, Indonesia)”. The ISRCTN registration number is: ISRCTN68253014 (www.isrctn.com).
Study protocol	The full study protocol of the above-mentioned study was published in "Trials" (Seuring et al., 2019). An additional substantially more detailed study protocol, describing the exact steps of this sub-study, was submitted to and approved by the Ethics Committee of the University of Göttingen and Universitas Syiah Kuala and is available for access upon request.
Data collection	The exact steps of data collection, including details of setting and time periods, are described in detail in the methods section of our manuscript. We kindly refer to this section.
Outcomes	The exact steps of outcome measurement, including pre-definitions and assessment, are described in detail in the methods section of our manuscript. We kindly refer to this section.
